# Quality Score Based Identification and Correction of Pyrosequencing Errors

**DOI:** 10.1371/journal.pone.0073015

**Published:** 2013-09-05

**Authors:** Shyamala Iyer, Heather Bouzek, Wenjie Deng, Brendan Larsen, Eleanor Casey, James I. Mullins

**Affiliations:** Department of Microbiology, University of Washington, Seattle, Washington, United States of America; New Jersey Institute of Technology, United States of America

## Abstract

Massively-parallel DNA sequencing using the 454/pyrosequencing platform allows in-depth probing of diverse sequence populations, such as within an HIV-1 infected individual. Analysis of this sequence data, however, remains challenging due to the shorter read lengths relative to that obtained by Sanger sequencing as well as errors introduced during DNA template amplification and during pyrosequencing. The ability to distinguish real variation from pyrosequencing errors with high sensitivity and specificity is crucial to interpreting sequence data. We introduce a new algorithm, CorQ (Correction through Quality), which utilizes the inherent base quality in a sequence-specific context to correct for homopolymer and non-homopolymer insertion and deletion (indel) errors. CorQ also takes uneven read mapping into account for correcting pyrosequencing miscall errors and it identifies and corrects carry forward errors. We tested the ability of CorQ to correctly call SNPs on a set of pyrosequences derived from ten viral genomes from an HIV-1 infected individual, as well as on six simulated pyrosequencing datasets generated using non-zero error rates to emulate errors introduced by PCR. When combined with the AmpliconNoise error correction method developed to remove ambiguities in signal intensities, we attained a 97% reduction in indel errors, a 98% reduction in carry forward errors, and >97% specificity of SNP detection. When compared to four other error correction methods, AmpliconNoise+CorQ performed at equal or higher SNP identification specificity, but the sensitivity of SNP detection was consistently higher (>98%) than other methods tested. This combined procedure will therefore permit examination of complex genetic populations with improved accuracy.

## Introduction

Massively parallel sequencing (MPS) or next generation (“next-gen”) sequencing technologies [1] allow for the generation of millions of sequence fragments (“sequence reads”) from a single specimen. These technologies have the potential to replace Sanger sequencing for many applications, including de-novo sequencing, re-sequencing and metagenomics [2], [3]. However, the promise of MPS has to be balanced with its caveats. Each MPS platform has a much higher rate of error compared to Sanger sequencing [1], [4], [5]. If the sample must be PCR-amplified prior to sequencing, the errors occurring during PCR are also present in the MP sequences and can be impossible to distinguish from real variation, except in cases when using random sequence tags coupled with oversequencing to generate consensus sequences from each amplicon [6], [7]. The 454/pyrosequencing platform results in uniquely high rates of overcalls and undercalls (resulting in erroneous insertions and deletions in the sequence reads) [1], [5]. Carry forward errors are also unique to pyrosequencing and are caused by leftover nucleotides in a sequencing well [1].

Error rates for the GS-FLX Titanium pyrosequencing technology have been estimated on an extensive dataset of Roche Corp. quality control DNA fragments and the sequences generated were found to have a mean error rate of 1.07% with errors showing a non-random distribution [5]. Error rates as high as 50% were reported in a few positions with the highest incidence of errors in homopolymer regions. Approximately 89% of the reads had some form of error. Thus, instead of removing reads with errors from downstream analysis, error correction methods are typically applied to an entire dataset to improve the overall accuracy of sequences, such as by filtering out regions of lower quality within reads [8].

Pyrosequencing errors have been corrected using two approaches: correcting the measured light intensities (called flowgrams) [9], [10], or correcting the generated sequences [11]–[18]. Methods to correct errors in pyrosequences using a Poisson or binomial probability model have traditionally assumed, incorrectly [1], [5], [8], that all base calls are of equal quality [11], [13], [16], [19]. Other error correction methods rely on comparing variants to an empirical control data set, mapping read segments to a consensus template and refining alignments locally [12]. Salmela and colleagues corrected errors by taking sequences sharing common k-mers and forming multiple alignments with these reads. The reads are then corrected based on a consensus sequence made from the resulting alignments [15]. Other correction methods employ phase (co-variation) information within reads to distinguish between real variation and systematic error [17]. These algorithms do not alter the flowgram data, instead they correct pyrosequencing errors on the translated bases. The program AmpliconNoise corrects pyrosequencing errors by clustering flowgrams and calculating the likelihood that each of the reads from these flowgrams was generated from a mixture of correct and incorrect sequences [9], [10]. Following this, an expectation-maximization algorithm is applied to the clusters to determine a true sequence for each cluster. AmpliconNoise has been used in determining microbial diversities of the human gut [20] and for viral population genetics [21].

Goals of the current study were twofold: to develop a widely applicable error correction method utilizing inherent base call quality and sequence context to correct pyrosequencing errors, and to make SNP calls based on the quality of the sequenced base. We developed a new error correction algorithm, CorQ that utilizes a multiple sequence alignment to map base qualities to the positions within the alignment. Reduction in base quality for an overcall/undercall error is detected by calculating the average drop in base quality between adjacent bases and the base in question, making use of quality scores from all the reads mapping to a position within the alignment. CorQ uses a set of sequences and associated quality files as a starting point for error correction. Carry forward errors that follow a specific pattern of single base insertions occurring after homopolymeric nucleotides are also identified and corrected as part of the program. In addition, we adapted CorQ to utilize sequence and associated quality files from other error correction and base recalibration algorithms [9], [10], [22]. We compared our method to other recently developed error correction and SNP calling methods, including CORAL [15], QuRe [18], SegminatorII [12] and V-Phaser [17]. We evaluated the sensitivity and specificity of these methods in identifying true SNPs found within a plasmid clone mixture of ten HIV-1 genomes derived from the blood plasma from one infected individual.

We also performed sensitivity and specificity analyses using CorQ and other error correction methods on six simulated pyrosequencing datasets. For these latter analyses we used two polymerase error rates 0.005, 0.01, selected based on experimental tests (Larsen *et al*., Manuscript under revision), to emulate errors generated during PCR amplification.

## Materials and Methods

### Pyrosequencing of HIV-1 Genomes

Ten HIV-1 genome sequences were PCR amplified, cloned and sequenced using the Sanger method from one HIV-1 infected individual [23]. We mixed these ten HIV-1 plasmid clones (GenBank accession numbers: JN024165–JN024168, JN024170–JN024173, JN024495 and JN024537) in equal proportion, linearized the DNA with a restriction enzyme, and performed pyrosequencing using the standard protocols provided in the GS-FLX Titanium Rapid Library preparation kit (454.com/products/gs-flx-system/index.asp).

### Generation of Simulated Pyrosequences

We generated a total of six additional simulated pyrosequencing datasets with Flowsim [24] using two starting configurations (Table S2). The first three datasets (Set 1a–c, Table S2) were generated using a single 1500 nt HIV-1 sequence as the starting template. Three simulation runs were conducted: The first had no additional SNP errors. The second and third had added SNP error rates of 0.005 and 0.01, respectively, set to approximate those generated during template PCR amplification, and were selected based on experimental tests (Larsen *et al*. Manuscript under revision). The templates for the fourth through sixth simulated pyrosequencing datasets (Set 2a–c, Table S2) were generated from a multiple sequence alignment of 28 previously published HIV-1 sequences [23]. A 1500 nt region was selected (alignment positions: 1–1522, File S1) and used as input for Flowsim. Again, three simulation runs were conducted: with no additional SNP errors, and with SNP error rates of 0.005 or 0.01.

### Error Correction with AmpliconNoise

AmpliconNoise [10] (version 1.24) was run on flowgrams using default settings. Error correction with AmpliconNoise suite of programs consists of two components, first clustering and correcting the flowgrams with AmpliconNoise followed by correcting PCR based errors with SeqNoise. In our preliminary evaluation we found that SeqNoise was computationally intensive yet it often failed on datasets larger than 20,000 reads and lacked important user definable parameters. Hence, we did not use the SeqNoise component for our subsequent analyses. The sequence and associated quality files obtained after AmpliconNoise flowgram correction were aligned with MOSAIK (http://bioinformatics.bc.edu/marthlab/Mosaik) using a sample-specific consensus sequence as reference. We adjusted the reference to query sequence mismatch parameter in MOSAIK to vary between 20–30%. This mismatch includes both SNP and indels and allowed mapping a greater number of reads to the reference sequence subsequently resulting in smaller loss of data.

### Read Filtering and Chimera Check

Sequences with ambiguous base calls (N) or less than 100 bases in length were removed, and we implemented an optional check to test for chimeric sequences. Chimeras are generated when sequences are amplified from a multi-template population [25] as well as naturally during HIV infection. The majority of in-vitro-generated chimeras arise due to incomplete primer extension during PCR [25]. To detect chimeras, we counted the number of SNP mismatches in a given read relative to the consensus sequence. In the CorQ analyses presented here we set this parameter to a default of 20% SNP mismatches between the consensus sequence and a given read to assign a sequence as a chimera, since this mismatch rate was optimal for chimera detection amongst several methods [26]. For analyzing sequences with inherently greater diversity, we recommend varying this parameter to better distinguish a sequence variant from artificially generated chimeric sequence.

### CorQ Implementation: Correcting Poor Quality Indel and Miscall Errors

CorQ uses the filtered sequence alignment file to correct indel and miscall errors. First, quality values are mapped to the bases in a multiple sequence alignment, and positions (Equation 1) with insertions and deletions in homopolymer and non-homopolymer regions are flagged. Two or more consecutive bases of the same kind are considered part of a homopolymer. In the flagged positions, the average base quality, Q_i_, of indel bases is estimated. The average base qualities of all called bases with associated qualities (A, G, T or C) in a non-indel position, Q_i-1_ and Q_i+1_, adjacent to flagged positions are also estimated. For each indel occurring after a homopolymeric or non-homopolymeric sequence, the average base quality difference, Q_reduction,i_, between the flagged and adjacent column is compared against a distribution of quality reductions across the entire alignment (Equation 1). Positions with greater than average base quality reduction are flagged for correction, handling single and multi-base indels in a similar manner. Indels present only in one read are also flagged for correction. Sequences containing flagged insertions are corrected by removing the incorrectly inserted base and sequences with flagged deletions are corrected by adding the consensus base. CorQ also creates an annotation file that tracks changes made to each corrected read.
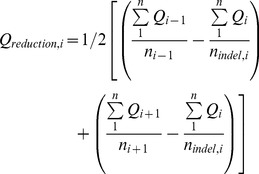
(1)


To identify and flag potential sequencing miscalls, the difference between the average base quality of all consensus bases, Q_i,consensus_, and average base quality of variant, Q_i,SNP_, bases is calculated for all positions with a SNP relative to the consensus sequence (Equation 2). The average reduction in SNP quality from the consensus is compared against a distribution of quality reductions for all SNPs and is flagged for correction if it is larger than the average in the distribution (Equation 2). The consensus character at that position then replaces a flagged SNP. Positions with a SNP present only within a single read in the dataset are also flagged for correction.
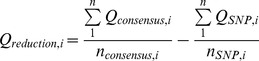
(2)


To accommodate uneven read coverage (number of reads mapping to each base) from the two different sequencing orientations, we implemented additional checks when correcting potential sequencing miscalls. We have made read coverage difference as one of the input parameters in CorQ to allow users to set a coverage difference threshold that best captures the observed read coverage differences. SNPs that fall within regions of the designated fold difference are marked but not corrected, as we cannot rule out the possibility that a detected SNP is not “true” simply due to lack of adequate reads mapping to that position.

We also implemented a method within CorQ to identify and correct carry forward errors. Carry forward errors occur when insufficient flushing between the flows results in leftover nucleotides in a well, resulting in signal peaks at the wrong position during the next base incorporation [1]. The presence of homopolymers increases the likelihood of this type of errors [1], [4]. Carry forward events cause single base insertions usually near, but not adjacent to homopolymer regions [4]. CorQ detects this specific pattern of single base insertions occurring after runs of homopolymeric nucleotides and flags them as carry forward errors if the inserted base is not the consensus at that position, and if it is the same base type as the preceding homopolymeric stretch. The flagged inserted bases are removed from reads.

### Comparison to other Error Correction Methods

We tested the sensitivity and specificity of CorQ to identify true SNPs within a dataset created by pyrosequencing ten HIV-1 genomes that had previously been sequenced, after cloning into plasmids, by the Sanger method, as well as the set of six simulated datasets. CorQ was tested against four other pyrosequencing error correction programs: CORAL [15], Segminator II [12], QuRe [18] and V-Phaser [17] and the flowgram correction method AmpliconNoise [10] using reads mapping to the three HIV genes *gag*, *env* and *nef*. All programs were run according to the default parameters recommended by the authors. We implemented CorQ on the following set of data files: a) uncorrected fasta and quality files, b) Flowgram corrected fasta and quality files (from AmpliconNoise) and c) files generated from the quality recalibration program Pyrobayes [22]. Pyrobayes uses data likelihoods and prior distributions to determine the Bayesian posterior probability of the correct number of bases given a measured incorporation signal [22] and results in a recalibrated base quality for each called base. We used the consensus of the Sanger sequences from the 10 viral genomes as the reference for generating multiple sequence alignments in all the above comparisons. V-Phaser results for simulated datasets were not included, as errors invariably occurred while running the program with these sequences that were not resolved in time for manuscript submission.

We also compared the performance of each program on indel attrition. The exact count of insertions and deletions are not obtained from the output from QuRe and SegminatorII, hence these programs were not included in this comparison.

## Results

**Figure 1:**
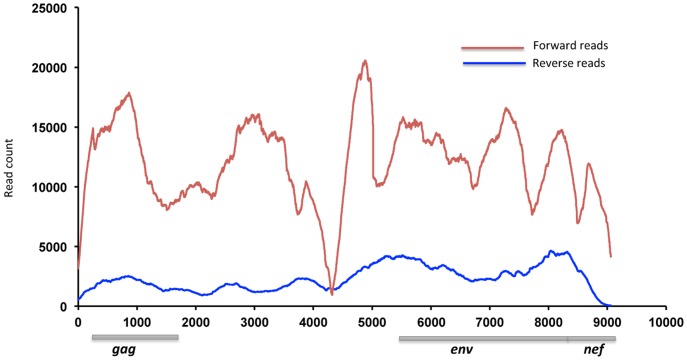
454 read coverage across the HIV-1 genome. Locations of the *gag*, *env* and *nef* genes evaluated in this study are shown. A total of 26,620 reads mapped to *gag*, 48,927 to *env* and 21,963 to the *nef* gene. Reads were aligned to a sample-specific consensus using MOSAIK (http://bioinformatics.bc.edu/marthlab/Mosaik).

Pyrosequencing of 10 HIV-1 genomes resulted in 26,620 reads mapping to *gag*, 48,927 reads mapping to *env* and 21,963 reads mapping to the *nef* genes. Read coverage for both sequencing orientations is shown in Figure 1. While these coverage maps are more uneven than typical pyrosequencing runs performed by us (unpublished results), they highlight an important concern for algorithms calling SNPs in regions of poor read coverage and for determining the actual depth of population sampling across a genome – coverage and depth vary across the target sequences, and thus are poorly summarized by a single measure.

**Figure 2:**
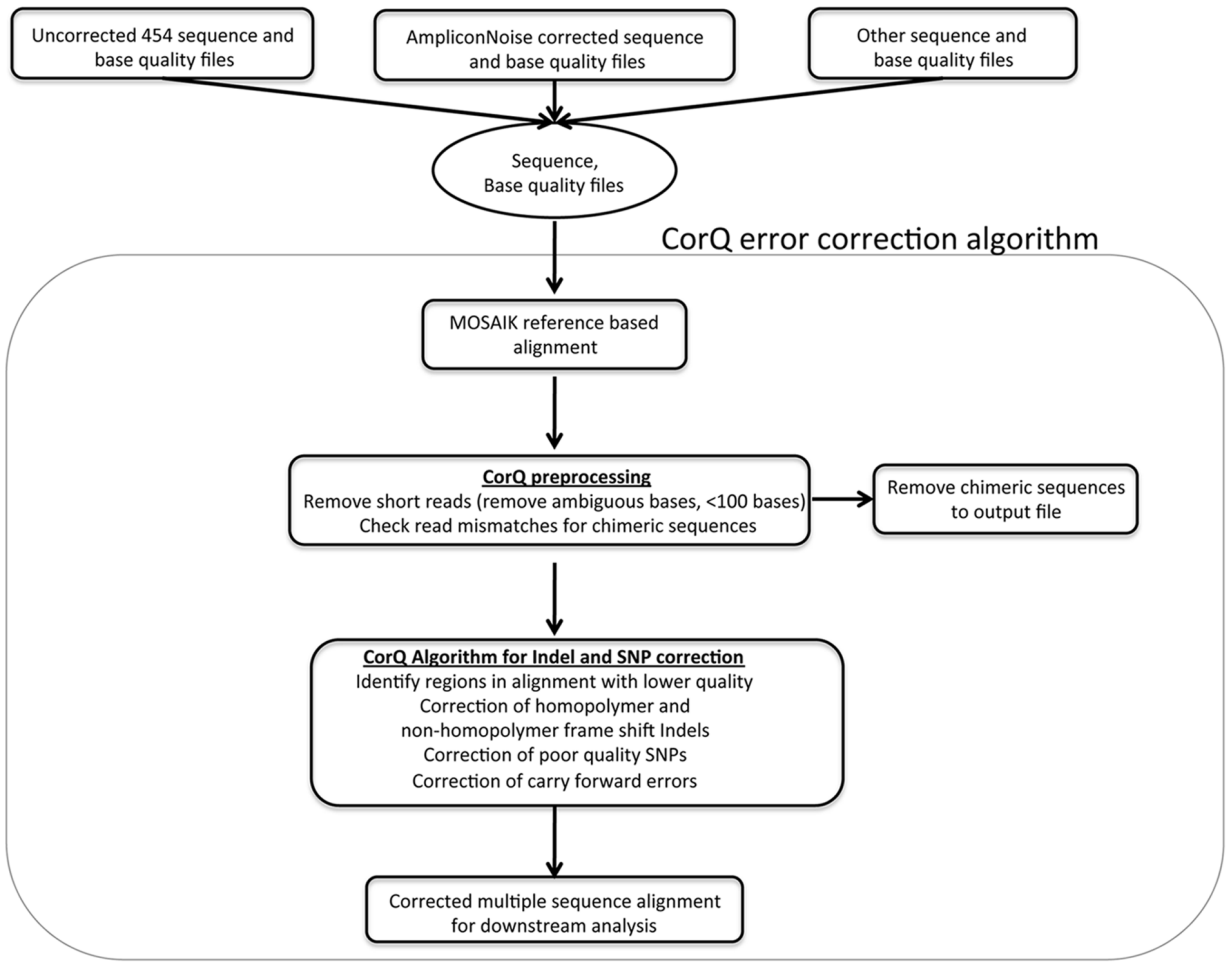
Overview of the CorQ 454 error correction methodology. The starting point for the CorQ algorithm is a set of sequence and base quality files. MOSAIK (http://bioinformatics.bc.edu/marthlab/Mosaik) is used for reference-based alignment. Positions with out-of-frame insertions and deletions (indels) are identified within the alignment and average base qualities are calculated for these regions (See Materials and Methods Equation 1). SNPs are similarly identified and called (See Materials and Methods Equation 2).

Figure 2 outlines the steps carried out by the CorQ error correction method. Following AmpliconNoise, a reference-guided multiple sequence alignment is generated with MOSAIK. Reads less than 100 bases and reads with ambiguous bases are removed as part of the preprocessing step. Short reads are generally a result of premature stops in strand synthesis or out-of-phase strand synthesis. These out-of-phase strands show early deterioration in signal quality, leading to shorter read lengths [1], [5]. Regions within the multiple sequence alignment with insertions and deletions are classified as occurring in homopolymer (a region with two or more consecutive nucleotides of the same type) or non-homopolymer regions. The average difference in base quality between an indel position and adjacent positions are then calculated. The rationale for this step is that in the event a base corresponds to a sequencing overcall or undercall, the quality of that base should be lower than the neighboring bases – CorQ measures this drop in base quality relative to the adjacent bases. A distribution of average base quality reductions across indel positions within the alignment is used to make error correction calls. We observed similar patterns of quality reductions across the three gene regions (Figure S1). This bolsters our hypothesis that erroneous bases have poorer quality in the reads that contain them, and that the base quality adjacent to an erroneous base should be higher in the majority of reads. This allows CorQ to identify regions with a drop in average base quality across an alignment.

**Figure 3:**
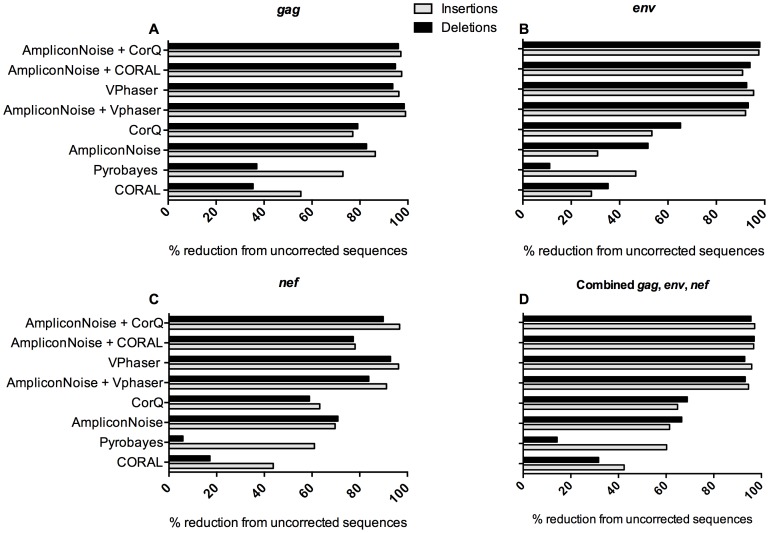
Attrition in indel counts after application of error correction methods. The percent reduction in number of indels within the HIV-1 ten-plasmid dataset compared to uncorrected sequences is presented. While Pyrobayes is not an error correction algorithm, but rather recalibrates quality values, the accuracy of recalibrated bases are meant to reflect overcalled and undercalled bases accurately. The % reduction in indels compared to uncorrected sequences is shown for *gag* (A), *env* (B) and *nef* (C), and all three genes combined (D).

Next, we compared the ability of CorQ and previously described algorithms for their ability to flag and correct sequencing overcalls and undercalls (Figure 3), using true indels observed within the Sanger sequences as an indicator of effectiveness. QuRe and SegminatorII programs did not output indel counts per position and hence we omitted these programs from this comparison. Vphaser run alone or the combination of AmpliconNoise flowgram correction followed by the CorQ algorithm on fasta and quality files reduced indel counts most effectively (95.4/96.7% reduction in *gag*, 95.3/94.7% in *nef* and 93/97% in *env*, respectively). CORAL, and Pyrobayes followed by CorQ did not result in a substantial reduction in erroneous indels (10–70%). Combinations of error correction methods performed better than applying a single correction method ranging from 93–97% reduction in indels. The combination of AmpliconNoise+CorQ and AmpliconNoise+CORAL performed better than other tested methods, achieving between 95–97% reduction in indels. Among the individual correction methods, VPhaser performed best, reducing indels by 92–96%.

**Figure 4:**
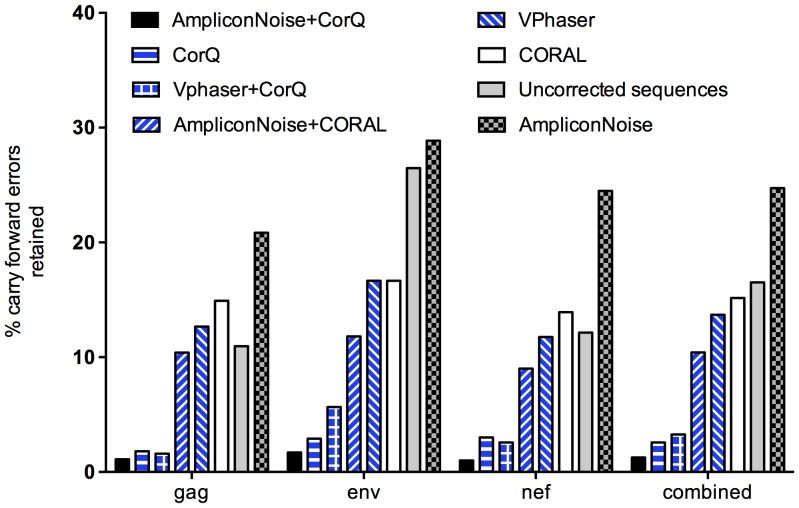
Carry forward errors retained after error correction. Raw uncorrected values and the percentage of carry forward errors retained after error correction is plotted for each of the three gene regions *gag*, *env*, *nef* and all the three genes combined.

CorQ also corrects carry forward errors [1] near homopolymeric regions. The percent carry forward errors retained within reads after application of error correction methods is shown in Figure 4. Carry forward errors present in raw uncorrected reads are shown for comparison. In the uncorrected reads, carry forward insertion errors make up about 10–30% of the total insertions errors observed. Flowgram error correction (AmpliconNoise) corrects homopolymeric overcall insertion errors to a greater extent than carry forward insertion errors, hence, 20–30% of the insertion errors are of carry forward type after flowgram correction. Vphaser and CORAL corrected carry forward errors better than AmpliconNoise, but still retained about 10–15% of these errors. The combination of AmpliconNoise+CORAL performed only slightly better than using CORAL alone, retaining ∼10% of carry forward errors. The combination of Vphaser correction followed by the carry forward correction segment of the CorQ program resulted in a further, substantial reduction in the number of carry forward errors compared to correction with Vphaser alone, retaining between 2–5% of these errors. The combination of AmpliconNoise+CorQ removed the most carry forward insertion errors, retaining only ∼2%.

The sensitivity and specificity of SNP identification was then compared for four pyrosequencing error correcting and variant calling algorithms within the *gag*, *env* and *nef* gene regions from our 10 HIV-1 genome dataset. Since the mixture was derived from ten whole genome plasmids mixed in equal proportion, the lowest observable valid SNP would be 10%, with SNPs calls in pyrosequences validated by comparison to variants identified in Sanger sequences [23]. A total of 28 SNPs in *gag* (1500 nt positions), 61 in *env* (2550 nt) and 21 in *nef* (681 nt) were compared. As shown in Table 1, the sensitivity of detection of variants was usually 97% or higher for most error methods, with the exception of the QuRe algorithm, which filters out regions with lower levels of coverage, and VPhaser when applied to the *nef* gene sequences. QuRe filtered out 3% of bases from correction for *gag* and *nef* but 33% of bases from correction in *env*. These filtered regions fell within areas of poor coverage, usually at the start of the gene. V-phaser had reduced sensitivity on the *nef* dataset (61%) due to a change of valid SNPs to consensus in a region with an in-frame 18 nt deletion present in 30% of the Sanger sequences. Changes to *gapwindow* size parameter (to match the gap size observed within the sequences) as part of the Vphaser correction program did not improve *nef* sensitivity. A combination of AmpliconNoise+CORAL also showed reduced sensitivity, with values falling lower than each of these correction methods used individually. CORAL corrects errors by forming a multiple sequence alignment and generating a consensus sequence from these alignments. It is possible that the low frequency of “real” SNPs that are seen after flowgram correction are removed in CORAL when consensus sequences are generated, thus leading to a higher incidence of false negatives and reducing sensitivity. Similarly, we observed a reduced sensitivity when we combined AmpliconNoise with Vphaser, with sensitivity values falling lower than each of these correction methods used individually. The combination of AmpliconNoise+CorQ consistently resulted in higher sensitivity than the other tested error correction methods used individually or in combination.

**Table 1:** Sensitivity and specificity of error correction algorithms in SNP variant calling.

Method	Sensitivity				Specificity			
	*gag*	*nef*	*env*	combined	*gag*	*nef*	*env*	combined
**Uncorrected 454 reads**	1	1	0.98	0.99	0.37	0.34	0.5	0.44
**CorQ**	1	1	0.98	0.99	0.79	0.86	0.94	0.88
**AmpliconNoise**	0.99	0.98	0.98	0.98	0.88	0.71	0.69	0.76
**AmpliconNoise+CorQ**	0.99	0.98	0.98	0.98	0.99	0.97	0.99	0.98
**Pyrobayes+CorQ**	0.97	1	0.98	0.98	0.78	0.7	0.78	0.77
**CORAL**	1	1	0.96	0.98	0.92	0.88	0.94	0.91
**AmpliconNoise+CORAL**	0.5	0.93	0.27	0.53	0.98	0.86	0.95	0.95
**QuRe**	0.96 (0.41)*	0.97 (0.61)*	0.98 (0.04)*	0.97 (0.11)*	0.97	0.92	0.99	0.96
**SegminatorII**	1	0.97	0.98	0.98	0.2	0.24	0.47	0.35
**VPhaser**	1	0.61	0.95	0.86	0.98	0.98	0.99	0.98
**AmpliconNoise+VPhaser**	0.54	0.25	0.41	0.38	1	0.99	1	0.99

Comparison of CorQ against other pyrosequence error correction and SNP calling algorithms. *gag*, *env* and *nef* gene regions were used to compare the sensitivity and specificity of various algorithms. Sensitivity measures the proportion of true SNPs present in the ten HIV-1 genomes, and correctly identified by the various SNP calling programs. Specificity measures the proportion of true negatives (positions in the gene regions that are invariant) that are correctly identified by the compared programs.

* Shown in parenthesis are values from QuRe when the poor coverage regions excluded from sensitivity analysis are included as false negatives.

With regard to specificity, the uncorrected reads had a high false positive rate (low specificity), and with the exception of SegminatorII each of the correction pipelines resulted in an increase in specificity. Repeated analyses with SegminatorII produced a high number of false positives, despite using a sample-specific consensus sequence as reference for the alignment and default settings recommended by the program authors. VPhaser alone, or flowgram correction (AmpliconNoise) in combination with CorQ, consistently produced the highest specificity for variant detection. Overall, combinations of error correction methods (AmpliconNoise+CORAL, AmpliconNoise+Vphaser and AmpliconNoise+CorQ) consistently exhibited between 86–100% specificity.

We also performed a test to assess the effects of read coverage differences across sequencing orientations on the sensitivity and specificity of CorQ to detect and correct SNPs. We used pyrosequences mapping to the ∼2500 nt *env* region from the ten HIV-1 plasmid clones for this comparison and ran the combination of AmpliconNoise+CorQ with 2-fold, 5-fold, 10-fold and 20-fold coverage differences as thresholds for SNP correction (Table S1). As expected with a lower read coverage difference threshold (2- or 5-fold), more positions were marked to be poor coverage regions – SNPs falling within these regions are not corrected, resulting in higher false positives (reducing specificity to 95%). With higher coverage difference thresholds (20-fold), more regions with SNPs are corrected, resulting in correction of real variation present within the sequences and giving more false negatives (reduced sensitivity to 95%). We therefore used a 10-fold coverage difference (98% sensitivity and 99% specificity) with CorQ to achieve a balance between sensitivity and specificity.

We tested the ability of error correction algorithms to reduce indel and substitution error rates in both homopolymeric and non-homopolymeric regions (Tables S3 and S4, respectively) on simulated pyrosequences generated with a single starting template (Sets 1a–c, Table S2). QuRe was not included in this analysis since it generates indel-removed haplotypes as the final result. SegminatorII was also excluded since it does not give indel information in the final results. The combination of AmpliconNoise+CORAL gives the highest reduction in substitution error rates for these simulated datasets. This mostly likely is a result of CORAL error correction whereby a regional consensus sequence is used to correct for low frequency variants. In the case where multiple sequencing templates are present, this correction method runs a risk of removing “true” low frequency variants (as we have shown with our sensitivity analyses), whereas in this case where only a single template was used for simulation, correction of low frequency variants is more efficient. Similar trends for indel and SNP error rate reduction was observed in homopolymeric and non-homopolymeric regions (compare Tables S3 and S4).

Lastly we also evaluated the sensitivity and specificity of SNP identification on simulated pyrosequencing datasets. We used the three simulated datasets (Sets 2a–c, Table S2) with multiple starting templates (28 templates) for this analysis. Prior Sanger sequencing had shown a total of 145 positions with SNPs within these 28 templates [23]. We did not include SegminatorII in this comparison since our previous analysis with this program (Table 1) had shown that it led to lower specificity than raw uncorrected reads. Vphaser was also excluded as errors in the program led to consistently failed runs (see Materials and Methods). When we compared the simulated sequences that lacked introduced SNP errors (Table 2), we observed very similar trends as observed with previous comparisons with the ten HIV-1 genome dataset (Table 1). As shown in Table 2, the sensitivity of detection was usually 95% or higher except in the combination of AmpliconNoise+CORAL that again showed a trend towards reduced sensitivity when combined. QuRe also showed reduced sensitivity when we included the poor coverage regions excluded by QuRe into our sensitivity calculations. When considering a balance between sensitivity and specificity, AmpliconNoise+CorQ performed the best amongst all the methods tested. As highlighted previously, PCR errors are harder for error correction algorithms to remove since these mutations are present within the sequencing templates. All error correction methods we tested on simulated pyrosequences with additional SNP errors added to emulate PCR errors fared poorly for the removal of false positives with the best being AmpliconNoise+CorQ, with a specificity of 40% (Table S5).

**Table 2:** Sensitivity and specificity of error correction algorithms in SNP variant calling in simulated pyrosequences (simulated datasets 2a–c).

Method	Flowsim simulated pyrosequences	
	Sensitivity	Specificity
**Uncorrected 454 reads**	0.99	0.15
**CorQ**	0.99	0.7
**AmpliconNoise**	0.99	0.89
**AmpliconNoise+CorQ**	0.99	0.95
**Pyrobayes+CorQ**	0.98	0.71
**CORAL**	0.95	0.88
**AmpliconNoise+CORAL**	0.2	0.99
**QuRe**	0.99 (0.44)*	0.98

Comparison of CorQ algorithm against other pyrosequence error correction and SNP calling algorithms. Simulated pyrosequences generated from 28 HIV-1 sequences as the starting template were used to compare the sensitivity and specificity of error correction algorithms. Sensitivity measures the proportion of true SNPs present within the HIV-1 templates used for simulation, and correctly identified as such by the various SNP calling programs. Specificity measures the proportion of true negatives (positions in the gene regions that are invariant) that are correctly identified as such by the compared programs.

* Values from QuRe are shown when the poor coverage regions excluded from sensitivity analysis are included as false negatives (shown in parenthesis).

## Discussion

We described a new pyrosequence error correction algorithm, CorQ that can identify and correct homopolymer and non-homopolymer indel errors, sequencing misincorporation errors and carry forward errors associated with homopolymeric regions. When applied to a control set of ten HIV-1 genomes (without PCR amplification), the combination of AmpliconNoise+CorQ reduced indel errors in the gene regions *gag*, *env* and *nef* by 94 to 97%. In addition to testing CorQ in combination with flowgram correction (AmpliconNoise) and base quality recalibration (Pyrobayes) programs, we also compared it to four recently published pyrosequencing variant callers, CORAL, QuRe, SegminatorII and V-Phaser. We found that when CorQ error correction is used on flowgram-corrected fasta and quality files produced by AmpliconNoise, we get consistently higher sensitivity and specificity of SNP detection. To tease apart the contribution of CorQ and AmpliconNoise, we ran the programs separately, and found that CorQ by itself improved SNP detection specificity to a range of 79% to 94%, whereas AmpliconNoise by itself improved specificity to a range of 69% to 88%, whereas uncorrected reads had a SNP detection specificity ranging from 34% to 50%. Combining AmpliconNoise and CorQ, however, consistently gave the highest combined SNP detection sensitivity and specificity amongst the error correction methods tested, with the specificity of VPhaser nearly equaling that of AmpliconNoise+CorQ. The combinations of AmpliconNoise+Vphaser and AmpliconNoise+CORAL while resulting in >86% specificity, had poor sensitivity ranging from 25%–93%.

The advantage of using AmpliconNoise+CorQ was most pronounced for the reduction carry forward errors. We also observed reductions in carry forward errors when we combined corrected files from Vphaser with CorQ, indicating that CorQ can be used in combination with other error correction programs to maximize the number of error free pyrosequences. We observed similar trends in sensitivity and specificity when we compared error correction methods on simulated pyrosequencing datasets. One caveat we observed in using AmpliconNoise is that it is computationally intensive, with computing time increasing exponentially on datasets over 20,000 reads, making this algorithm impractical for large datasets without extensive computational resources. Furthermore, since AmpliconNoise relies on iterative clustering, we have observed that the frequencies of low-level SNPs do not correlate well with the frequencies found within uncorrected reads for sequences generated through amplicon sequencing on the Roche 454 platform (unpublished results). We therefore recommend use of AmpliconNoise for library pyrosequencing only, as described here.

CorQ takes read coverage into account when making SNP calls, particularly in regions in which there is a large discrepancy between the number of reads obtained in one sequencing orientation compared to the other. Other pyrosequencing error correction methods we tested here do not explicitly address read coverage variation across the target sequence or in different sequencing orientations. We addressed this by requiring a SNP to be present in both orientations. We also made read coverage difference threshold an input parameter for CorQ so that users can use the fold coverage that appropriately represents the data they are analyzing. We settled on a default setting of 10-fold coverage difference after initial tests showed this to achieve a good balance between SNP detection sensitivity and specificity. Thus, in regions with over a 10-fold difference in read coverage across sequencing orientations, SNPs are not corrected (by CorQ) due solely to inadequate information. While this criterion does not address all possible scenarios of read coverage across sequenced positions, we have observed that most regions with coverage discrepancies also tend to have inadequate or lack of reads in one of the sequencing orientations (unpublished observations).

As expected, none of programs evaluated were able to correct SNPs present in sequences as a result of misincorporation events occurring during PCR of the template preparation, unless, in the case of CorQ, these SNPs also had reduced base quality. This makes identification of SNP errors as a result of PCR amplification challenging by any method as shown by our error correction tests run on simulated pyrosequences with typical PCR error rates applied.

We selected HIV-1 sequences as templates for generating additional simulated pyrosequences as this technology has become widespread in studying HIV-1 genomes. The genetic diversity of HIV-1 found within an infected individual in chronic infection is comparable to the global genetic variation seen in the influenza virus [27]. The most prominent source of HIV-1 mutation is error prone nucleic acid synthesis during replication, with rates estimated in the range of 1.4×10^–5^ errors per base pair, per replication cycle [28]. Viral diversity also differs in different genes and with the length of infection. The diversity of a viral population within an infected individual starts low immediately after infection but increases during the course of infection at a rate of 1% (within the *env* region) reaching up to 15% or more in long term infected individuals [29]. This extent of diversity makes pyrosequencing both a useful and challenging tool to study HIV-1. The information gleaned from pyrosequences thus has to be judged carefully for errors from both the sequencing methodology and PCR amplification.

CorQ lists frequencies of SNPs and outputs a multiple sequence alignment that can be used for downstream analysis of a variety of datasets, including microbial communities. Other error correction methods such as QuRe and V-Phaser that were tested here also generate reconstructed haplotypes that can be useful in studying microbial communities. Researchers interested in studying diverse microbial communities can use the information provided here to make decisions on selecting the right set of error correction tools. While we have tested CorQ on data derived from pyrosequencing, this algorithm is general enough to be applied to sequences generated from other high throughput platforms that generate both sequence and associated quality files, making it a method with widespread applications in variant detection. Perl scripts that implement each step in the CorQ pipeline are available for download at: http://mullinslab.microbiol.washington.edu/publications/iyer_2012/.

## Supporting Information

Figure S1 **Average reduction in base quality for indels found in homopolymer and non-homopolymer regions.** Reduction in base quality was measured as the average difference in quality between flagged positions with indels and the adjacent columns (See Materials and Methods, Equation 1). Base qualities from uncorrected sequences (raw 454), and sequences corrected with AmpliconNoise and Pyrobayes are shown for indels found in non-homopolymer regions (length of 1) and varying homopolymer lengths. Reduction in base quality is shown for indels within *gag* (A), *env* (B), *nef* (C) and the three genes combined (D).
(TIFF)

Table S1 **Effect of varying coverage threshold on sensitivity and specificity of SNP variant calling.** AmpliconNoise+CorQ error correction was used on pyrosequences mapping to the *env* region (∼2500 nt) from the ten HIV-1 genome control dataset. Different fold coverage values were used as input parameters in CorQ. Sensitivity and specificity of SNP variant detection within this region is calculated for each fold coverage value.
(DOCX)

Table S2 **Average number of reads and average read length for simulated pyrosequences.** Two sets of simulated pyrosequences generated using Flowsim are shown here. The first set (Set 1a, b and c) is comprised of simulated reads generated using a single 1500 nt HIV-1 sequence as the starting template. The second set (Set 2a, b and c) is comprised of simulated reads generated using a 1500 nt region located within 28 HIV-1 sequences as starting templates. Simulations were done without additional SNP errors (1a, 2a) and with two different SNP error rates, 0.005 and 0.01 (1b,c and 2b,c).
(DOCX)

Table S3 **Comparison of insertion, deletion and substitution error rates in homopolymeric regions after error correction on simulated pyrosequences.** Simulated reads were generated using Flowsim using a single 1500 nt HIV-1 sequence as the starting template (Simulated datasets 1a–c). Average insertion, deletion and substitution error rates within homopolymeric regions are shown after correction with no additional SNP errors, and SNP error rates of 0.005 and 0.01.
(DOCX)

Table S4 **Comparison of insertion, deletion and substitution error rates in non-homopolymeric regions after error correction on simulated pyrosequences.** The simulated reads were generated in Flowsim using a single 1500 nt HIV-1 sequence as the starting template (Simulated datasets 1a–c). Average insertion, deletion and substitution error rates within non-homopolymeric regions are shown after correction with no additional SNP errors, and SNP error rates of 0.005 and 0.01.
(DOCX)

Table S5 **Sensitivity and specificity comparison of error correction and SNP calling algorithms on simulated pyrosequences.** Simulated datasets 2a–c was used to compare the sensitivity and specificity of error correction algorithms. Sensitivity measures the proportion of true SNPs present within the HIV-1 templates, and correctly identified as such by the various SNP calling programs. Specificity measures the proportion of true negatives (positions in the gene regions that are invariant) that are correctly identified as such by the compared programs. Note that QuRe failed when used on simulated pyrosequences generated with a SNP error rate of 0.005. * Values from QuRe are shown when the poor coverage regions were excluded from sensitivity analysis and when these regions are included as false negatives during analysis (the latter values are shown in parenthesis).
(DOCX)

File S1 **Sequences for generating simulated pyrosequences.**
(TXT)
