# Supplementary figures and images for: Quality Score Based Identification and Correction of Pyrosequencing Errors
Source: PLoS One. 2013 Sep 5;8(9):e73015. doi: 10.1371/journal.pone.0073015 (PMC3764156; doi:10.1371/journal.pone.0073015)

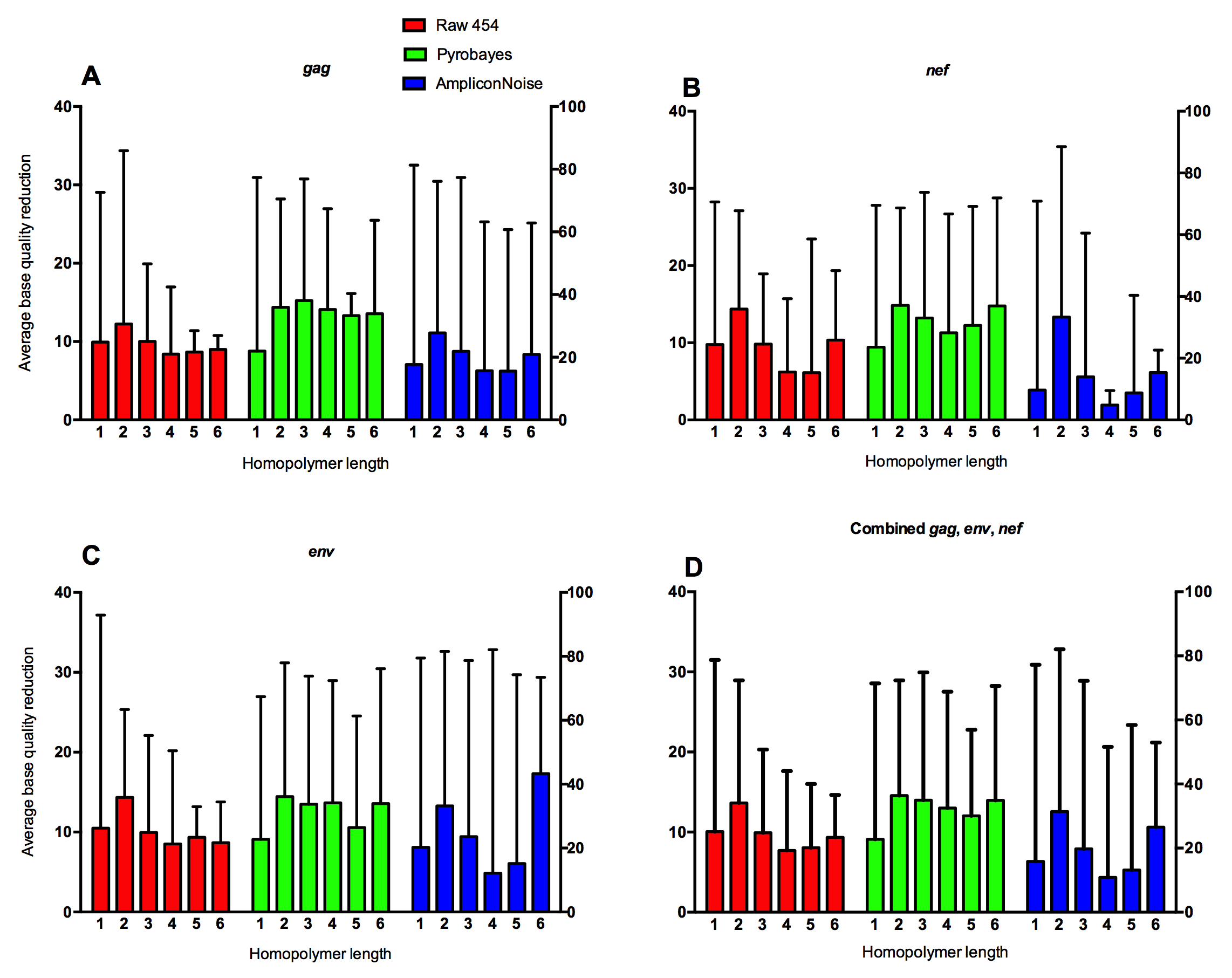

Supplement: Figure S1 — Average reduction in base quality for indels found in homopolymer and non-homopolymer regions. Reduction in base quality was measured as the average difference in quality between flagged positions with indels and the adjacent columns (See Materials and Methods, Equation 1). Base qualities from uncorrected sequences (raw 454), and sequences corrected with AmpliconNoise and Pyrobayes are shown for indels found in non-homopolymer regions (length of 1) and varying homopolymer lengths. Reduction in base quality is shown for indels within gag (A), env (B), nef (C) and the three genes combined (D). (TIFF) [file pone.0073015.s001.tiff]
