# Supplementary material for: Quality Score Based Identification and Correction of Pyrosequencing Errors
Source: PLoS One. 2013 Sep 5;8(9):e73015. doi: 10.1371/journal.pone.0073015 (PMC3764156; doi:10.1371/journal.pone.0073015)
Supplement: Table S1 — Effect of varying coverage threshold on sensitivity and specificity of SNP variant calling. AmpliconNoise+CorQ error correction was used on pyrosequences mapping to the env region (∼2500 nt) from the ten HIV-1 genome control dataset. Different fold coverage values were used as input parameters in CorQ. Sensitivity and specificity of SNP variant detection within this region is calculated for each fold coverage value. (DOCX) [file pone.0073015.s002.docx]

**Supplementary Table S1**

| **Coverage threshold** | **Sensitivity** | **Specificity** |
| --- | --- | --- |
| 2 fold coverage | 0.98 | 0.95 |
| 5 fold coverage | 0.98 | 0.96 |
| 10 fold coverage | 0.98 | 0.99 |
| 20 fold coverage | 0.95 | 0.99 |
