# Supplementary material for: Quality Score Based Identification and Correction of Pyrosequencing Errors
Source: PLoS One. 2013 Sep 5;8(9):e73015. doi: 10.1371/journal.pone.0073015 (PMC3764156; doi:10.1371/journal.pone.0073015)
Supplement: Table S2 — Average number of reads and average read length for simulated pyrosequences. Two sets of simulated pyrosequences generated using Flowsim are shown here. The first set (Set 1a, b and c) is comprised of simulated reads generated using a single 1500 nt HIV-1 sequence as the starting template. The second set (Set 2a, b and c) is comprised of simulated reads generated using a 1500 nt region located within 28 HIV-1 sequences as starting templates. Simulations were done without additional SNP errors (1a, 2a) and with two different SNP error rates, 0.005 and 0.01 (1b,c and 2b,c). (DOCX) [file pone.0073015.s003.docx]

Supplementary Table S2

| **Simulated pyrosequencing set** | **Reads generated** | **Average read length (nt)** |
| --- | --- | --- |
| **Set 1a (No additional SNP errors)** | 36,000 | 494 |
| **Set 1b (SNP error rate: 0.005)** | 33,000 | 427 |
| **Set 1c (SNP error rate: 0.01)** | 48,000 | 428 |
| **Set 2a (No additional SNP errors)** | 98,800 | 423 |
| **Set 2b (SNP error rate: 0.005)** | 98,880 | 422 |
| **Set 2c (SNP error rate: 0.01)** | 98,000 | 423 |
