# Supplementary material for: Quality Score Based Identification and Correction of Pyrosequencing Errors
Source: PLoS One. 2013 Sep 5;8(9):e73015. doi: 10.1371/journal.pone.0073015 (PMC3764156; doi:10.1371/journal.pone.0073015)
Supplement: Table S3 — Comparison of insertion, deletion and substitution error rates in homopolymeric regions after error correction on simulated pyrosequences. Simulated reads were generated using Flowsim using a single 1500 nt HIV-1 sequence as the starting template (Simulated datasets 1a–c). Average insertion, deletion and substitution error rates within homopolymeric regions are shown after correction with no additional SNP errors, and SNP error rates of 0.005 and 0.01. (DOCX) [file pone.0073015.s004.docx]

Supplementary Table S3

|  | **No additional SNP errors** | | | **SNP error rate: 0.005** | | | **SNP error rate: 0.01** | | |
| --- | --- | --- | --- | --- | --- | --- | --- | --- | --- |
|  | **Insertion** | **Deletion** | **Substitution** | **Insertion** | **Deletion** | **Substitution** | **Insertion** | **Deletion** | **Substitution** |
| **Uncorrected** | 0.004 | 0.003 | 0.00032 | 0.006 | 0.005 | 0.0025 | 0.006 | 0.005 | 0.0045 |
| **AmpliconNoise** | 0.003 | 0.002 | 0.00013 | 0.004 | 0.0045 | 0.0023 | 0.004 | 0.0044 | 0.0042 |
| **CorQ** | 0.0035 | 0.002 | 0.00015 | 0.005 | 0.0042 | 0.0018 | 0.0051 | 0.0044 | 0.0031 |
| **Pyrobayes + CorQ** | 0.003 | 0.0028 | 0.00026 | 0.0056 | 0.0048 | 0.0019 | 0.0055 | 0.0047 | 0.0038 |
| **AmpliconNoise + CorQ** | 0.0018 | 0.001 | 0.00003 | 0.002 | 0.0014 | 0.0002 | 0.002 | 0.0018 | 0.00042 |
| **CORAL** | 0.0008 | 0.0009 | 0.0 | 0.0008 | 0.0004 | 0.0002 | 0.0007 | 0.0009 | 0.0002 |
| **AmpliconNoise + CORAL** | 0.0003 | 0.0004 | 0.0 | 0.0006 | 0.0002 | 0.00001 | 0.0003 | 0.0004 | 0.00002 |
