# Supplementary material for: Quality Score Based Identification and Correction of Pyrosequencing Errors
Source: PLoS One. 2013 Sep 5;8(9):e73015. doi: 10.1371/journal.pone.0073015 (PMC3764156; doi:10.1371/journal.pone.0073015)
Supplement: Table S4 — Comparison of insertion, deletion and substitution error rates in non-homopolymeric regions after error correction on simulated pyrosequences. The simulated reads were generated in Flowsim using a single 1500 nt HIV-1 sequence as the starting template (Simulated datasets 1a–c). Average insertion, deletion and substitution error rates within non-homopolymeric regions are shown after correction with no additional SNP errors, and SNP error rates of 0.005 and 0.01. (DOCX) [file pone.0073015.s005.docx]

Supplementary Table S4

|  | **No additional SNP errors** | | | **SNP error rate: 0.005** | | | **SNP error rate: 0.01** | | |
| --- | --- | --- | --- | --- | --- | --- | --- | --- | --- |
|  | **Insertion** | **Deletion** | **Substitution** | **Insertion** | **Deletion** | **Substitution** | **Insertion** | **Deletion** | **Substitution** |
| **Uncorrected** | 0.0008 | 0.0006 | 0.0001 | 0.0023 | 0.0027 | 0.002 | 0.0023 | 0.0027 | 0.0038 |
| **AmpliconNoise** | 0.0 | 0.0001 | 0.0001 | 0.0021 | 0.0022 | 0.0018 | 0.0021 | 0.0022 | 0.0036 |
| **CorQ** | 0.0 | 0.0001 | 0.00001 | 0.00098 | 0.001 | 0.0009 | 0.001 | 0.0015 | 0.001 |
| **Pyrobayes + CorQ** | 0.00001 | 0.0004 | 0.00009 | 0.0011 | 0.0019 | 0.0017 | 0.0018 | 0.002 | 0.0026 |
| **AmpliconNoise + CorQ** | 0.0 | 0.00005 | 0.00004 | 0.0001 | 0.0009 | 0.0006 | 0.0008 | 0.0009 | 0.0009 |
| **CORAL** | 0.0 | 0.00002 | 0.0 | 0.00007 | 0.0001 | 0.00002 | 0.00007 | 0.0002 | 0.0002 |
| **AmpliconNoise + CORAL** | 0.0 | 0.0 | 0.0 | 0.0 | 0.0 | 0.0 | 0.00004 | 0.0 | 0.00003 |
