# Supplementary material for: Quality Score Based Identification and Correction of Pyrosequencing Errors
Source: PLoS One. 2013 Sep 5;8(9):e73015. doi: 10.1371/journal.pone.0073015 (PMC3764156; doi:10.1371/journal.pone.0073015)
Supplement: Table S5 — Sensitivity and specificity comparison of error correction and SNP calling algorithms on simulated pyrosequences. Simulated datasets 2a–c was used to compare the sensitivity and specificity of error correction algorithms. Sensitivity measures the proportion of true SNPs present within the HIV-1 templates, and correctly identified as such by the various SNP calling programs. Specificity measures the proportion of true negatives (positions in the gene regions that are invariant) that are correctly identified as such by the compared programs. Note that QuRe failed when used on simulated pyrosequences generated with a SNP error rate of 0.005. * Values from QuRe are shown when the poor coverage regions were excluded from sensitivity analysis and when these regions are included as false negatives during analysis (the latter values are shown in parenthesis). (DOCX) [file pone.0073015.s006.docx]

Supplementary Table S5

| **Method** | **SNP error rate: 0.005** | | **SNP error rate: 0.01** | |
| --- | --- | --- | --- | --- |
|  | **Sensitivity** | **Specificity** | **Sensitivity** | **Specificity** |
| **Uncorrected 454 reads** | 1 | 0.09 | 1 | 0.03 |
| **CorQ** | 1 | 0.19 | 1 | 0.04 |
| **AmpliconNoise** | 1 | 0.16 | 1 | 0.07 |
| **AmpliconNoise + CorQ** | 1 | 0.71 | 1 | 0.40 |
| **Pyrobayes + CorQ** | 1 | 0.16 | 1 | 0.07 |
| **CORAL** | 0.89 | 0.15 | 1 | 0.04 |
| **AmpliconNoise + CORAL** | 0.80 | 0.79 | 0.95 | 0.32 |
| **QuRe** | - | - | 0.55 (0.26)* | 0.96 |
